# Increased Incidence of Pyogenic Liver Abscess in a Midwest System With Emphasis on Rural Impact

**DOI:** 10.7759/cureus.21477

**Published:** 2022-01-21

**Authors:** Meghan Grassel, Douglas Yim, Jackson Shriver, Tanner Redlin

**Affiliations:** 1 Family Medicine, University of South Dakota Sanford School of Medicine, Sioux Falls, USA; 2 Interventional Radiology, Johns Hopkins Health System, St. Petersburg, USA; 3 Interventional Radiology, University of South Dakota Sanford School of Medicine, Sioux Falls, USA

**Keywords:** : liver abscess, water infrastructure, rural healthcare, hepatic abscess, pyogenic liver abscess

## Abstract

Objective: To assess if hepatic abscess incidence in a Midwest cohort was higher in rural areas compared to metropolitan areas in relation to water infrastructure.

Materials and methods: All cases of hepatic abscesses from Jan 1, 2016 through Dec 31, 2019 at Avera McKennan Hospital in Sioux Falls, South Dakota (SD), were retrospectively collected. Chart review was completed for each case for risk factor analysis. Microbiology cultures and patient demographic data were collected including age, gender, hometown, and ethnicity. Risk factors assessed included a history of abdominal surgery, gallbladder disease, sepsis, diverticulitis, cancer, and diabetes. The incidence of hepatic abscesses was calculated using the Poisson rate test and confidence interval equation. Averages of each risk factor were calculated. Finally, hometown was utilized to create a heatmap of disease burden and compared to the density of private wells.

Results: Our data yielded 116 confirmed adult hepatic abscesses between 2016 and 2019. The corrected incidence per 100,000 hospitalized patients per year is 95.66. The Poisson exact probability P-value was <0.01. Rural areas had a higher per capita incidence of abscesses and higher density of private wells.

Conclusions: The incidence of hepatic abscesses is higher than national averages in this single-center study of Avera McKennan Hospital. Demographics, especially geographic location, play an important role in abscess rates. Rural location may be affecting the incidence of hepatic abscesses, explaining the much higher than expected incidence in this study. Infrastructure could be a contributing factor as much of the rural area is reliant on untreated groundwater.

## Introduction

Pyogenic liver abscesses are a significant medical problem with high morbidity and mortality. The incidence ranges from 8 to 20 abscesses per 100,000 hospital admissions. Multiple case series have shown that the incidence of hepatic abscesses has been increasing in recent years [1-4]. On average, the patient diagnoses with a hepatic abscess is between 50-60 years old. Other demographic factors have not been reported to affect incidence.

We aimed to assess if hepatic abscess incidence in a Midwest cohort was higher in rural areas compared to metropolitan areas in relation to water infrastructure.

## Materials and methods

Inclusion criteria 

All cases of hepatic abscesses from 2016 through 2019 at Avera McKennan Hospital in Sioux Falls, South Dakota (SD), were retrospectively collected by screening ICD-10 codes and the pictures archiving and collection system (PACS) for hepatic abscesses with IRB approval number 2019.069/100635 from Avera Research Institutes. The PACS system stores imaging and appointment information for patients seen with imaging findings of hepatic abscess. One hundred sixteen unique patients were identified. Patient medical ID numbers were assigned to ensure the uniqueness of the data.

Study design

Patient demographic data collected included: age, gender, hometown, and ethnicity. Risk factors assessed included a history of abdominal surgery, gallbladder disease, sepsis, diverticulitis, cancer, and diabetes. Following the collection of patient city and state of residence from the electronic medical record, the corresponding county of residence was collected and attached to previously recorded patient data. County-specific population data was recorded based on the 2018 US census. Documented cases were then totaled on a per county basis and averaged on a per year basis for 2016-2019. This number was then adjusted to a per capita incidence rate. Total cases by county and per capita were plotted over a map of SD using the Microsoft Excel 3D mapping tool. The number of domestic wells was then quantified using the Water Well Completion Reports from the SD Government website. A heat map was generated with this data for comparison as well.

Statistical analysis

After confirming the presence of a hepatic abscess, we completed a chart review for risk factor analysis. The total number of hospitalized patients from 2016-2019 at the Avera McKennan campus was compiled and used as a reference for the total hospitalized patient population. The incidence of hepatic abscesses was then calculated using the Poisson rate test and confidence interval equation using the exact Poisson method. We used the known incidence of hepatic abscesses as 20 cases per 100,000 hospitalized patients [1-4]. A confidence interval was then calculated using the Garwood chi-square at 95%. The alpha used was 0.05. After determining significance, we calculated percentages of each risk factor and microbiology cultures.

## Results

Our data yielded 116 confirmed adult hepatic abscess cases at Avera McKennan between 2016 and 2019. There were 121,266 total hospitalized adults in that same period. The corrected incidence per 100,000 hospitalized patients per year is 95.66, 95% confidence interval of 80-120, and was significantly different from national estimates with a P-value of <0.01. Baseline characteristics are listed below in Table 1.

**Table 1:** Baseline characteristics

Characteristic	Total (N=116)
Age	
Age Group	
18-34	4 (3.4%)
35-49	18 (15.5%)
50-80	81 (69.8%)
>80	13 (11.2%)
Sex	
Male	73 (62.9%)
Female	43 (37.1%)
Race	
White	95 (81.9%)
African American	4 (3.4%)
American Indian	10 (8.6%)
Asian	2 (1.7%)
Hispanic	2 (1.7%)
Other	2 (1.7%)
Unknown	1 (0.9%)

**Figure 1:**
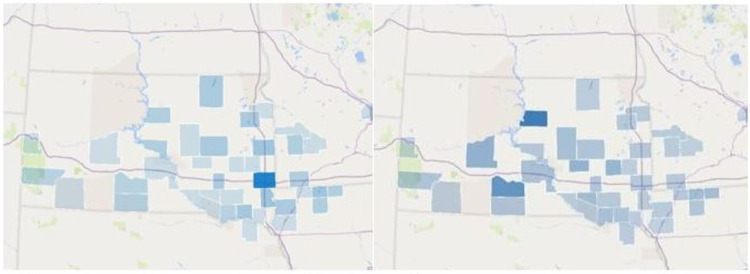
Hepatic abscess cases per county (left) vs hepatic abscess cases per county per capita (right) Darker blue = increased density

When considering total cases of known hepatic abscesses, the counties of Minnehaha, SD (28), Dickinson, Iowa (IA) (6), Brookings, SD (6), Lincoln, SD (6), Yankton, SD (5), Beadle, SD (4), Davison, SD (4), Codington, SD (4), Sioux, IA (4), and Brown, SD (4) were the ten counties with the highest total number of cases as shown in Figure 1. 

These counties also tended to be centered in larger population centers and accounted for eight of the largest eleven counties within South Dakota. However, when considering the incidence rate on a per capita basis, the results were much different. The counties of Potter, SD (33.98), Mellette, SD (24.49), Haakon, SD (13.03), Jerauld, SD (20.43), Miner, SD (11.30), Dickinson, IA (8.74), Douglas, SD (8.52), Emmet, IA (8.11), Hand, SD (7.66) and Bon Homme, SD (7.16) were found to have the highest incidence rate per 100,000 residents (Figure 1). These counties, on average, were much smaller and rurally isolated than the counties with the highest gross incidence rate. With the exception of Dickinson, IA, and Emmet, IA, these counties each had less than 5,000 residents. Risk factors for hepatic abscesses followed national trends as shown in Table 2. Prior abdominal surgery and cancer were the largest predisposing risk factors to forming a hepatic abscess in our study.

**Table 2:** Risk factors for hepatic abscess

Characteristic	Total (N=116)
Abdominal Surgery	71 (61.2%)
Cancer	47 (40.5%)
Diabetes Hx	43 (37.1%)
Diverticulitis Hx	22 (19.0%)
Sepsis Hx	49 (42.2%)
Type of Surgery	
Appendectomy	12 (10.3%)
Cholecystectomy	31 (26.7%)
Colorectal	14 (12.1%)
Gastric Bypass	2 (1.7%)
Gynecological	13 (11.2%)
Hernia Repair	9 (7.8%)
Liver	7 (6.0%)
Pancreas	1 (0.9%)
Urological Surgery	3 (2.6%)
Whipple	6 (5.2%)
Other	3 (2.6%)
Type of Cancer	
Blood Dyscrasias	5 (4.3%)
Cholangiocarcinoma	3 (2.6%)
Colon	7 (6.0%)
Liver	4 (3.4%)
Lung	4 (3.4%)
Pancreas	14 (12.1%)
Prostate	4 (3.4%)
Other	8 (6.9%)

Of the abscesses that a microbe was successfully isolated: Streptococcus intermedius (16), Klebsiella pneumoniae (9), Enterococcus (6), Escherichia coli (E. coli) (5), Fusobacterium (4), Staphylococcus epidermidis (3), and Candida (3) were most common. Most abscesses did not grow organisms or were not set for culture. There was one case of Entamoeba histolytica.

## Discussion

Several conditions can increase the risk of hepatic abscesses. The major identifiable cause of liver abscesses is ascending cholangitis [1,5,6]. Pylephlebitis from diverticulitis, prior surgery, medical conditions that can lead to systemic infection, and sepsis have all been identified as potential contributors to hepatic abscess development as well [1,6]. Sometimes no discernable direct cause is found and is then classified as cryptogenic. Systemic illnesses such as diabetes mellitus and cancer can predispose patients to cryptogenic abscess [1,6-8]. After determining the most likely cause, abscesses are categorized as infectious, malignant, or iatrogenic to guide antimicrobial treatment [6].

Geographic location has been previously implicated globally as a risk factor for abscess incidence. Rural areas have more liver abscesses in India compared to metropolitan areas [9]. Rural residents have less access to infrastructure compared to urban residents. The Safe Water Drinking Act only applies to public water access such as city water or rural water. Around 15% of the US population relies on private wells for water meaning they are not covered by the Safe Water Drinking Act and thus not required to be tested for water quality. The rate of private water use is likely proportionally higher in rural areas [10,11]. South Dakota only requires wells to be tested for water quality at the time they are drilled or dug and does not require action based on results. These residents that are reliant on private well water may be at higher risk for water contamination from chemical and microbial contamination [10,11]. Groundwater conditions can be affected by landfills, mining operations, chemical spills, pesticide and fertilizer application, and waste from animals [12]. Coliform and E. coli levels are usually not a source of infectious disease, however, these organisms are used as a metric of bacterial burden for other pathogens that are not as easily tested [11]. Untreated groundwater in smaller water treatment facilities has higher rates of coliform and E. coli detection indicating that rural water sources are at higher risk for bacterial burden as well [13]. Increased coliform and E. coli detection has been shown to correlate with increased gastrointestinal infection for residents using that water source [11].

The incidence of hepatic abscesses in our Midwest center was significantly higher at 95 cases per 100,000 hospital admissions than the expected national incidence of 20 cases per 100,000 hospital admissions. The only other upper Midwest study on pyogenic liver abscesses completed in Olmstead county, Minnesota (MN) had only 72 cases over 35 years, whereas our study had 116 cases over five years [4]. The center in our study is in a more rural area compared to the Olmstead County study but has similar demographics otherwise [4]. Olmstead county has a population of around 158,000 [4]. While this is smaller than the population of South Dakota, Olmstead county is entirely served by one health care facility. Whereas, South Dakota's population is divided between multiple health care providers. The number of patients served in all of Olmstead county and Avera McKennen are similar. is unclear why there is an increased incidence in our region. Proposed reasons for the increased incidence in a more rural state like South Dakota include but are not limited to: rural areas are more likely to have resource scarcity and may be less able to diagnose and treat disease efficiently, inflated cases from patients traveling large distances for specific care at our center, and influence of rural infrastructure such as water quality [14-19]. More frequent surgical treatment of systemic diseases like cancer and increased rates of surgical procedures could be reasons for a national increase in rates of hepatic abscesses; however, this would not fully explain this study’s deviation from other studies [2,4,6,7,14].

The demographic information collected in this study is largely concordant with prior national data [4,6,8]. Although this study’s cohort was predominantly Caucasian, this is consistent with the racial distribution in South Dakota and surrounding areas. The cases were mostly adults over 50, which is expected based on national data1. Our risk factors followed national trends as well.

As expected, counties with the largest populations had the highest total cases of abscesses. However, when viewed as a per capita, rural counties had an increased incidence (Figure 1). Regardless of the etiology, rural areas seem to have a much higher incidence of abscesses in this study. Accordingly, providers in rural areas may need to have a higher degree of suspicion for hepatic abscesses based on the increased incidence in rural areas in this study.

**Figure 2:**
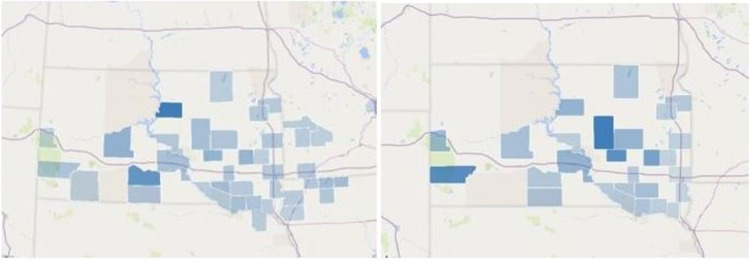
Hepatic abscess cases per county per capita (left) vs South Dakota well density per county (right) Darker blue = increased density

Many patients in rural areas rely on well water, which could play a previously unrecognized role in the development of hepatic abscesses [20]. It is difficult to infer correlation with well water as rural residents may rely on private wells or rural water for drinking water. Residents may have a private well but not rely on it for drinking water. Similarly, residents could have access to rural water and choose to utilize well water. Some residents may have multiple private wells for one family unit, further skewing the data. Some rural counties particularly rely on well water for livestock and may have a disproportionate number of private wells because of livestock production. Domestic well density was used as a crude metric, assuming that as the number of wells increased, private well use may also increase. However, this data should be used cautiously and as a springboard for further research, rather than a causative finding. When domestic well density was compared to hepatic abscess cases per capita, similar trends were seen. There are higher rates of domestic wells in rural counties, just as there are higher rates of hepatic abscesses in rural counties (Figure 2). Raw data is below in Table 3. 

**Table 3:** Gross and per capita incidence of hepatic abscess and domestic wells

County	Population	Annual Occurrence	Annual rate per individual	Case Count	Annual Cases per 100,000 residents	Wells	Wells per 100,000 residents
Hand	3,262	0.25	0.0001	1	7.66	955	29276.52
Custer	8,726	0.5	0.0001	2	5.73	2059	23596.15
Jerauld	2,043	0.25	0.0001	1	12.24	462	22613.80
Miner County	2,213	0.25	0.0001	1	11.30	366	16538.64
Potter	2,207	0.75	0.0003	3	33.98	236	10693.25
Lyman	3,821	0.25	0.0001	1	6.54	393	10285.27
Beadle	18,883	1	0.0001	4	5.30	1940	10273.79
Lawrence	25,741	0.25	0.0000	1	0.97	2132	8282.51
Hutchinson	7,380	0.25	0.0000	1	3.39	487	6598.92
Haakon County	1,918	0.25	0.0001	1	13.03	124	6465.07
Todd	10,283	0.5	0.0000	2	4.86	518	5037.44
Brown	39,316	1	0.0000	4	2.54	1932	4914.03
Bon Homme	6,980	0.5	0.0001	2	7.16	328	4699.14
Douglas County	2,935	0.25	0.0001	1	8.52	131	4463.37
Mellette	2,042	0.5	0.0002	2	24.49	84	4113.61
Grant	7,147	0.25	0.0000	1	3.50	277	3875.75
Charles Mix	9,338	0.25	0.0000	1	2.68	268	2869.99
Davison	19,790	1	0.0001	4	5.05	557	2814.55
Hughes	17,650	0.75	0.0000	3	4.25	308	1745.04
Codington	28,015	1	0.0000	4	3.57	483	1724.08
Yankton	22,869	1.25	0.0001	5	5.47	336	1469.24
Brookings	35,232	1.5	0.0000	6	4.26	365	1035.99
Lake County	13,057	0.25	0.0000	1	1.91	90	689.29
Clay	14,041	0.75	0.0001	3	5.34	110	783.42
Lincoln	58,807	1	0.0000	4	1.70	189	321.39
Minnehaha	192,876	7	0.0000	28	3.63	282	146.21

However, the only non-rural county in this study was Minnehaha, SD. It is difficult to infer correlation with limited metropolitan counties for comparison. Globally pyogenic liver abscess incidence is much higher than the US [19]. Differences in water infrastructure globally have been cited as a reason for this discrepancy [19]. We postulate that this could translate to the increased incidence in our rural cohort within the US as well. Private wells are more likely to have higher levels of coliform and E. coli when tested [21]. A study in Wisconsin found that nearly half (47%) of private wells had water quality outside of the parameters set by the Safe Water Act [21]. Private water quality issues could be a contributing factor to the increasing incidence of pyogenic liver abscesses in rural areas.

Additionally, patients in rural areas could be delaying care for conditions like cholecystitis because of a lack of convenient access to healthcare facilities. Individuals in these communities may not have the ability to travel to a larger medical center to have a procedure done and increase their risk for liver abscesses from ascending cholangitis due to delayed care [16-18]. Interestingly, Streptococcus intermedius was the most common pathogen isolated from microbiologic studies. Prior studies have not shown this to be a frequent organism causing hepatic abscesses [1,7,14]. However, many abscesses in this study did not isolate a specific organism and microbiological data in previous studies has varied by study [1,5,7,14]. In this study, E. coli and Klebsiella pneumoniae were the second and third most commonly isolated organisms following national trends. 

There are a few potential drawbacks to this study. Our data is not entirely complete as the study was limited to one of the two healthcare systems in our area. Counties not served by Avera were not represented on our distribution map. There is a possibility that abscesses could be treated more frequently at one of the two systems causing possible inflation of incidence. Further study involving more comprehensive data is needed to draw complete conclusions. Additional investigation into causes of increased incidence of pyogenic hepatic abscess in rural communities is also needed to help combat this potential health disparity.

## Conclusions

The incidence of hepatic abscesses is higher in this rural study compared to national incidences. We postulate that infrastructure could be a contributing factor, as many rural residents are reliant on untreated groundwater. The overall incidence of hepatic abscesses tends to vary by region, however, our study's incidence is much higher than incidences reported in other literature. Further investigation into possible predisposing variables such as the use of untreated water, water quality metrics, and health care disparities is necessary to understand reasons for disparities in rural incidence compared to metropolitan areas.
